# Enhancing Multiple Jets in Electrospinning: The Role of Auxiliary Electrode

**DOI:** 10.3390/nano8100768

**Published:** 2018-09-28

**Authors:** Yu-Ke Wu, Zong-Jie Li, Jie Fan, Zhao-Peng Xia, Yong Liu

**Affiliations:** 1 National Joint Engineering Research Center of High Performance Fibers and Textile Composites, Tianjin Polytechnic University, Tianjin 300387, China; 13388096568@163.com (Y.-K.W.); lpllzj@126.com (Z.-J.L.); fanjie@tjpu.edu.cn (J.F.); 2 School of Textiles, Tianjin Polytechnic University, Tianjin 300387, China

**Keywords:** electrospinning, nanofibers, auxiliary electrode, jet number

## Abstract

An auxiliary electrode introduced in traditional spinneret electrospinning is an effective and powerful technique to improve the production rate of nanofibers. In this work, the effects of the arrangement of auxiliary electrode, applied voltage, injection speed, and the distance between the electrode tip and the spinneret tip (ESD) on the jet number and the morphology of polyvinyl alcohol (PVA) nanofibers were investigated systematically. The results showed that the number of jets firstly increased and then decreased with the increase of applied voltage and ESD, respectively, while increasing with the injection speed in both the auxiliary electrode in the vertical position and parallel position. The average nanofiber diameter decreased with increasing of applied voltage and injection speed, but decreasing in ESD in these two positions. The numerical simulation results revealed that the auxiliary electrode primarily influenced the electric field intensity in the spinning area. This work provides a deep understanding of multiple jets in electrospinning.

## 1. Introduction

Electrospinning is a viable way to obtain ultrafine and nanofibers from polymer solutions of both natural and synthetic [1,2,3]. During electrospinning, polymer solution is charged and deformed under high electric field. Then, under the driving force of electric field, a polymer droplet is stretched from hemispheres to a cone at the end of capillary tube, that is, a Taylor cone [4]. When the electric field is large enough, a jet will be formed from the surface of the Taylor cone. The jet liquid, being charged, and will be deposited in the receiving collector after high-speed stretching in the electric field. Finally, the polymer nanofibers will be formed. Electrospun nanofibers have attracted great attention in the past decade due to their unique advantages such as high surface-to-volume ratio, adjustable porosity of fiber structures, and the flexibility to spin into a variety of shapes and sizes [5,6,7], which has huge potential for many applications such as filtration [8,9,10], biomedical materials [11,12], energy generation and storage [13,14], drug release [15,16,17], coatings [18], and sensors [19,20].

There is generally a single jet ejected from a Taylor cone in traditional spinneret electrospinning, and the production rate of nanofibers is very low [21]. In order to improve the yield of nanofibers, the increase in the number of spinnerets in electrospinning was commonly employed [22]. However, static mutual interference between adjacent spinnerets caused unevenness or perforation on nanofiber membranes [23].

Needleless electrospinning has been proved as an efficient technique to improve nanofiber productivity [24,25]. The electrospinning system produced fine nanofibers on a much larger scale [26]. However, the preparation of nanofibers in needleless electrospinning still has some problems, such as a complicated electrospinning system, high voltage power supplies, and rapid solvent evaporation, as well as high costs [27].

In order to combine the advantages of spinneret electrospinning and needleless electrospinning, an auxiliary-electrode electrospinning, which could produce multiple jets (2–12), was proposed in our previous work [28]. This novel method provides a new and powerful approach for the mass production of nanofibers. Recently, the influence of polymer solution properties on the jets was investigated systematically. However, there is lack of a deep understanding of the role of the auxiliary electrode on the jet number and the morphologies of nanofibers.

In this study, electrospinning with the auxiliary electrode (AE) with different arrangements (parallel or vertical to the spinneret) under different conditions such as applied voltage, and distance between electrode tip and spinneret tip was employed to investigate jet motion, number of jet, and fibers morphology. COMSOL Multiphysics 5.0 software was used to provide a possible formation mechanism of multiple jets formation.

## 2. Experimental Section

### 2.1. Materials and Methods

Polyvinyl alcohol (PVA, Mw = 84,000–89,000 g/mol), was purchased from Taiwan Changchun Oil Chemical Co. Ltd. (Taiwan, China). The PVA powder was dissolved in distilled water to form solution of 6 wt% concentration [29]. The solutions were stirred at 80 °C in a water bath for 5 h until they became homogeneous. The solution was used without further purification. During the electrospinning process, parameters included the distance between the electrode tip and the spinneret tip (ESD) of 1, 2, 3, and 4 cm, injection speeds of 1, 1.5, 2, and 2.5 mL/h, and the applied voltage from 0 to 9 kV. The distance between the spinneret tip and collector (SCD) was set to 15 cm in all the experiments.

### 2.2. Electrospinning Setup

**Figure 1:**
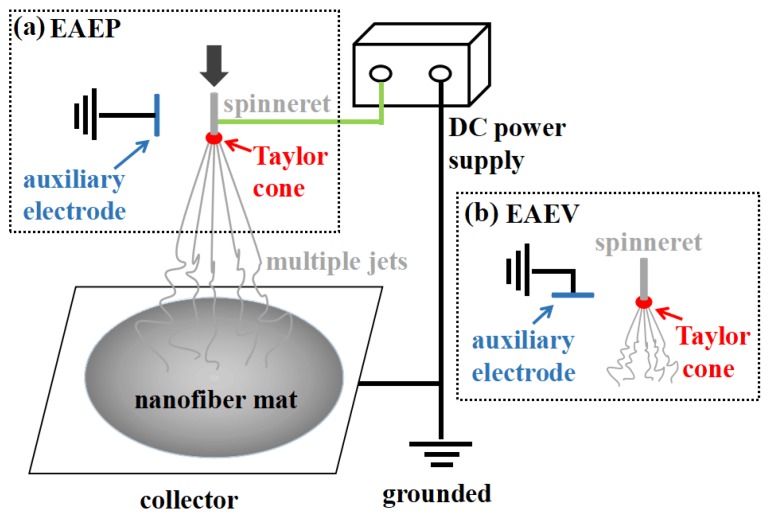
Schematic of multi-jet electrospinning process using an auxiliary electrode (AE) (**a**) Electrospinning with AE at parallel position (EAEP); (**b**) Electrospinning with AE at vertical position (EAEV).

The schematic diagram of the electrospinning with AE in this study is shown Figure 1. It consisted of a syringe pump (LSP02-1B, Baoding Longer Pump company, Baoding, China) and needle spinneret (stainless steel hollow needle, with the outside diameter (OD) = 1.2 mm, the inner diameter (ID) = 0.79 mm), a grounded auxiliary electrode (stainless steel solid needle, OD = 0.5 mm) parallel or vertical to the spinneret, and a high voltage supply (DW-P803-4ACF2, Tianjin Dongwen Company, Tianjin, China). All experiments were carried out at room temperature.

The fiber morphology was investigated using a scanning electron microscope (SEM) (Hitachi S4800 FESEM, Hitachi Co. Ltd., Tokyo, Japan). Before observation, the membranes were sputtered with gold. Image analysis software Nano measure 1.2 was used to measure the diameters of fibers based on SEM images, and more than 100 fibers were counted to calculate the average fiber diameter. Both the trajectory and the number of electrospun jets were recorded by a digital camera (FinePix HS11, FUJIFILM, Tokyo, Japan). The average number of jets was recorded five times by the digital camera. The electric field of the spinning area was simulated by the COMSOL Multiphysics 5.0 software (COMSOL Inc., Stockholm, Sweden).

## 3. Results and Discussion

### 3.1. Electrospinning and Electric Field Analysis

**Figure 2:**
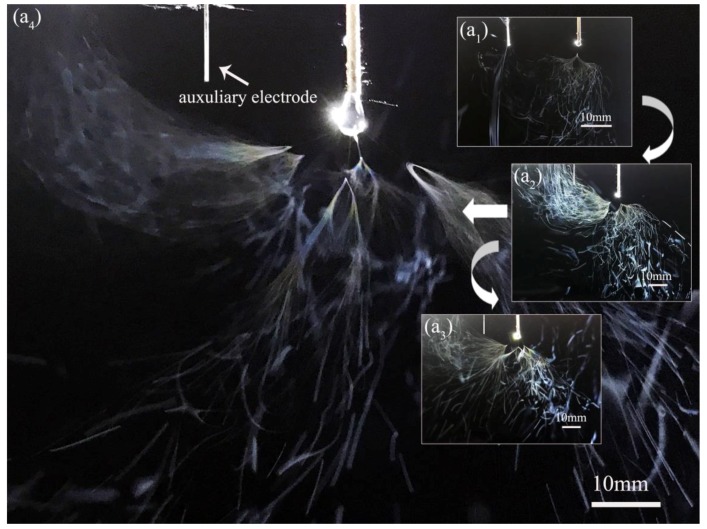
Images of jets produced by electrospinning with AE at parallel position (EAEP). (**a_1_**) 1 jet at 4.34 kV; (**a_2_**) 2 jets at 4.85 kV; (**a_3_**) 3 jets at 5.58 kV; (**a_4_**) 5 jets at 6.5 kV.

**Figure 3:**
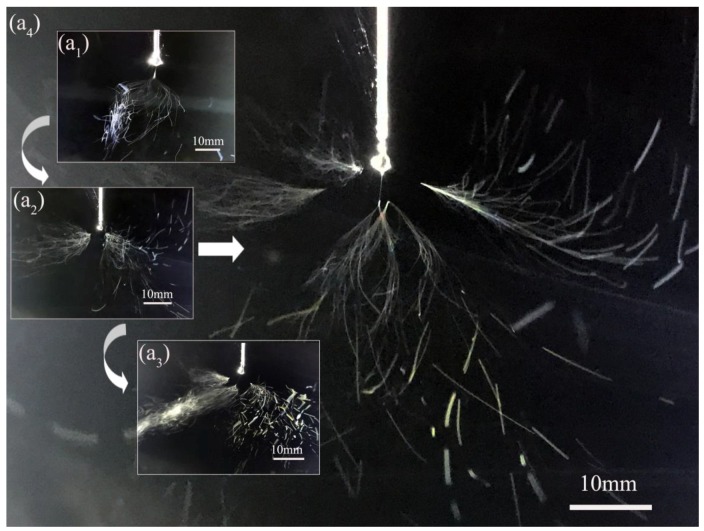
Images of jets produced by electrospinning with AE at vertical position EAEV. (**a_1_**) 1 jet at 4.68 kV; (**a_2_**) 2 jets at 5.2 kV; (**a_3_**) 3 jets at 6.12 kV; (**a_4_**) 5 jets at 6.84 kV.

During the electrospinning process, the polymer solution was charged and deformed under high electric field. When the applied voltage reached the threshold value, a jet ejected from the Taylor cone. For the electrospinning with auxiliary electrode at parallel position (EAEP), no jet formed when the applied voltage was lower than 4.34 kV for PVA/water solution. When the applied voltage was over such threshold voltage, the primary jet generally appeared first from the Taylor cone (Figure 2(a_1_)), followed by the jet whipping. When the auxiliary electrode was at the vertical position (EAEV), the threshold applied voltage was 4.68 kV (Figure 3(a_1_)), higher than that of the EAEP, in this electrospinning process. As the voltage increased, 2, 3, and 5 jets were produced by EAEP at 4.85, 5.58, and 6.5 kV (Figure 2(a_2_–a_4_)) and EAEV at 5.2, 6.12, and 6.84 kV (Figure 3(a_2_–a_4_)), showing that the applied voltage of EAEP was lower than that of EAEV when producing the same number of jets. Such a phenomenon may be attributed to the different electric field distribution around the spinneret of EAEP and EAEV.

**Figure 4:**
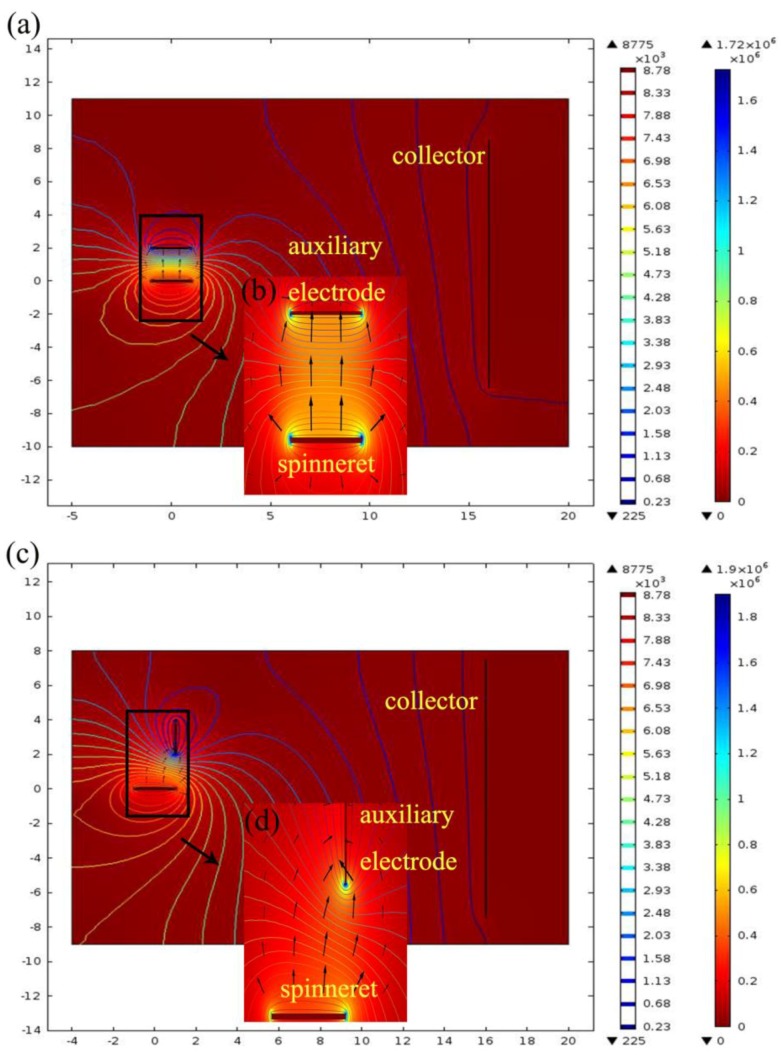
Simulation of electric field distribution in spinning area: (**a**,**b**) EAEP; (**c**,**d**) EAEV.

**Figure 5:**
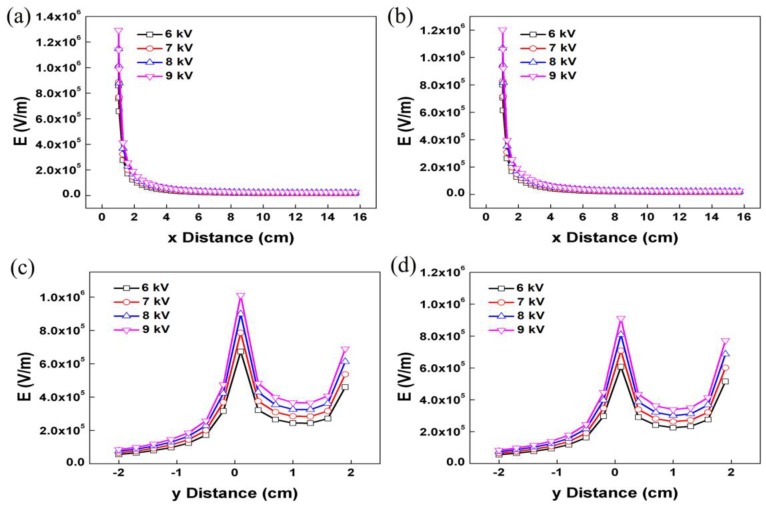
Graphs of electric field magnitude versus axial distance from the spinneret tip to the collector in x direction (**a**) EAEP; (**b**) EAEV; y direction (**c**) EAEP; (**d**) EAEV.

For a better understanding of the experimental findings, the electric field distribution of EAEP and EAEV were calculated and compared by finite element method (FEM) COMSOL Multiphysics 5.0 software. In the present simulation work, the parameters were as follows: a 2 cm long and 0.05 mm diameter auxiliary electrode parallel or vertical to a 2 cm length spinneret with 0.08 mm diameter: ESD of 2 cm; an applied voltage of 9 kV; and a spinneret-collector distance (SCD) of 15 cm. Figure 4a,c show the electric field distribution of EAEP and EAEV, it can be clearly found that the electric field with high intensity is restricted to a relatively small domain, and there is a noticeable jump of the electric field intensity. The arrows indicate the direction of the electric field, and their length is proportional to the strength of the electric field at that position (Figure 4b,d). The simulation results showed that the electric field intensity along the x-axis (Ex) and y-axis (Ey) in EAEP (Figure 5a,c) and EAEV (Figure 5b,d) were compared under different applied voltages (6, 7, 8, and 9 kV). It can be seen that the auxiliary electrode at parallel position can enhance the electric field strength at the spinneret tip, e.g., Ex = 1.2 × 10^6^ V/m in EAEV, but Ex = 1.29 × 10^6^ V/m in EAEP at 9 kV. This could enable the threshold voltage of producing jets in EAEP lower than that of EAEV, and eject more jets in EAEP than those in EAEV, as demonstrated in Figure 2 and Figure 3. More jets generally means higher production rate of nanofibers in electrospinning.

### 3.2. Effect of AE Position on Fiber Morphology under Different Applied Voltage

**Figure 6:**
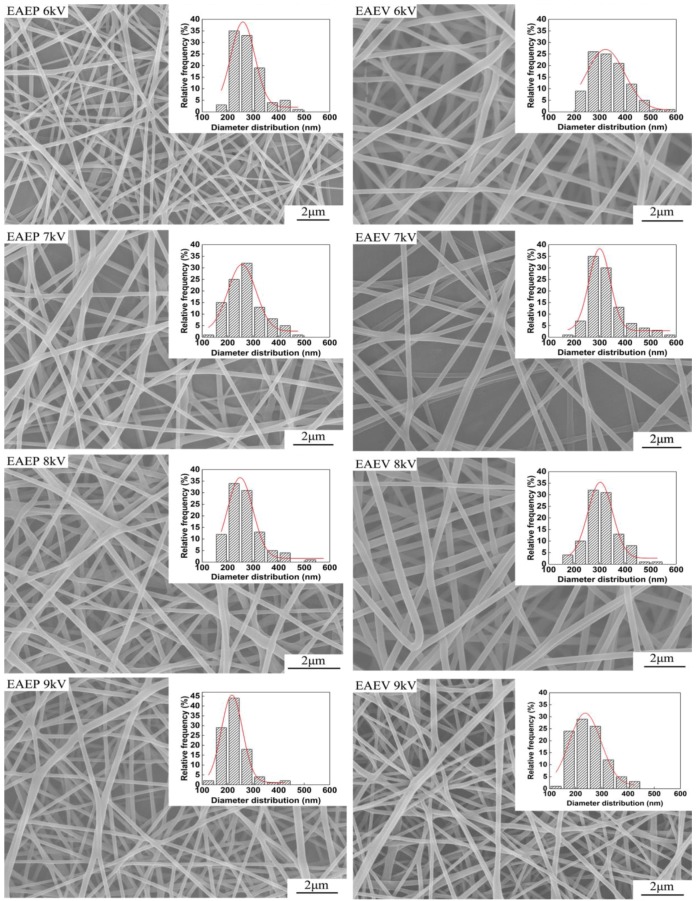
SEM images of electrospun nanofibers produced by EAEP and EAEV under different applied voltages.

**Figure 7:**
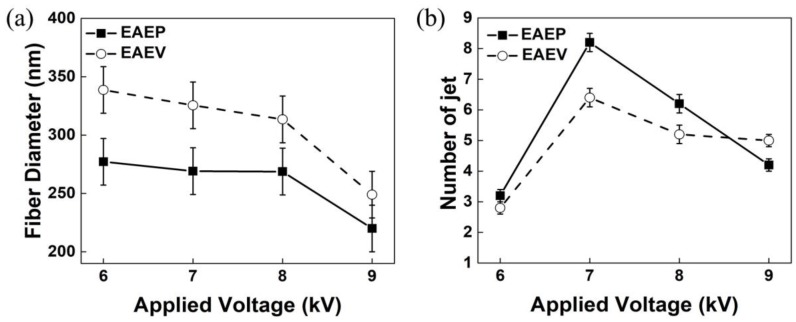
(**a**) Average fiber diameter vs. applied voltage and (**b**) Jet number vs. average voltage in EAEP and EAEV.

To investigate the effect of AE position on fiber morphology, the applied voltage was set as 6, 7, 8, and 9 kV. Other conditions were kept at ESD of 2 cm, injection speed of 2 mL/h, and SCD of 15 cm. The SEM images of the nanofibers obtained from both systems are shown in Figure 6. The morphologies of the nanofibers obtained from the two systems are similar, having typical non-wovens characteristics of round fibers and porosity. With an increase of the applied voltage (6–9 kV), the average fiber diameter decreased in both systems (Figure 7a). For example, the average fiber diameter decreased from 277 nm to 220 nm in EAEP and from 338 nm to 249 nm in EAEV as applied voltage increased from 6 to 9 kV. Additionally, the average diameters of nanofibers in EAEP were less than those in EAEV under the same applied voltage. The reason might be that there is higher electric field intensity in EAEP than that in EAEV, as shown in simulation results of Figure 5. The AE could change rapidly the original electric field distribution around the spinning area and cause a high electric field intensity gradient between the spinneret and AE. As a result, higher electric fields can generate stronger electric field forces, facilitate jet formation, promote solution jet whipping, and reduce fiber diameter [30].

The average numbers of jets were recorded by the digital camera and are shown in Figure 7b. When applied voltage was 6 and 7 kV, the average jet number of EAEP (3.2 and 8.2) was larger than that of EAEV (2.8 and 6.4). The reason might be that the electric field force in EAEP is stronger than that in EAEV. When the applied voltage continuously increased to 8 kV, the electric field force of EAEP is too large to reduce the distance between jets [31], thus resulting in fewer jet numbers, 6 jets in this experiment. For the EAEV system, when applied voltage reached 8 and 9 kV, the electric field force of EAEV was also too large to reduce jet numbers to 5. It means the jet numbers firstly increased and then decreased with the increase of applied voltage in these two systems.

### 3.3. Effect of AE Position on Fiber Morphology under Different Injection Speed

**Figure 8:**
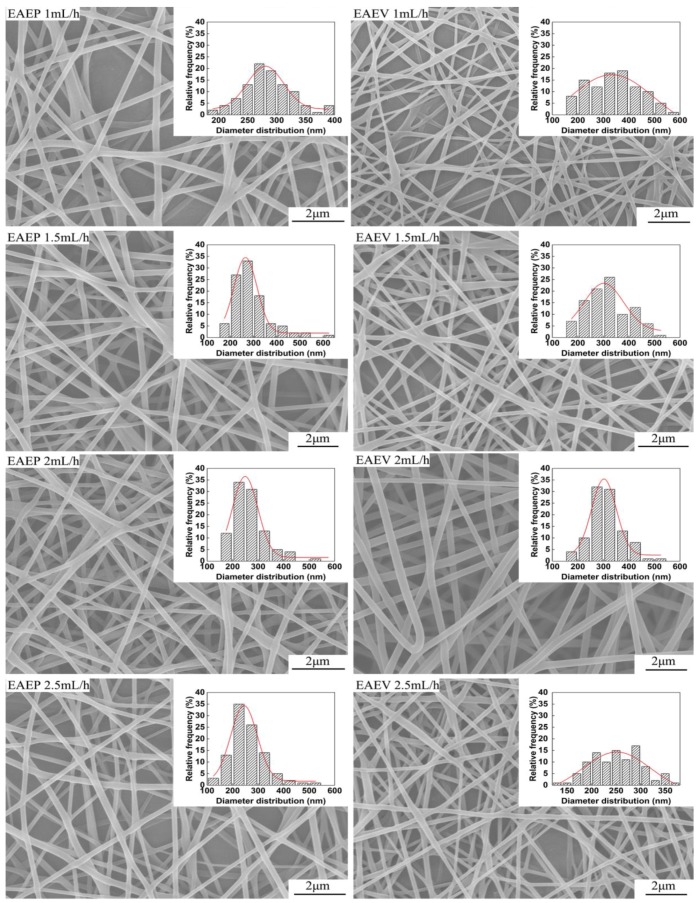
SEM images of electrospun nanofibers produced by EAEP and EAEV with different injection speeds.

**Figure 9:**
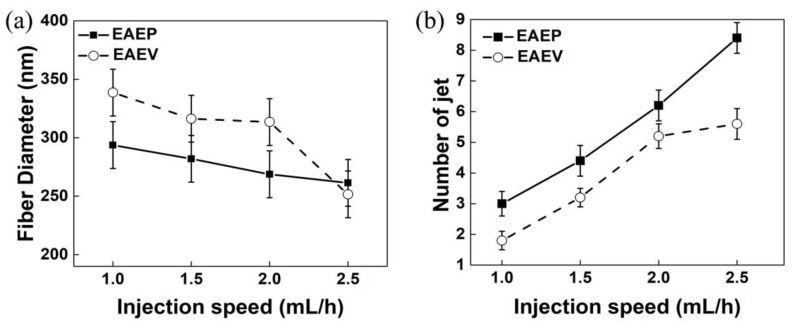
(**a**) Average fiber diameter vs. injection speed and (**b**) Jet number vs. injection speed in EAEP and EAEV.

Figure 8 shows SEM images of nanofibers obtained from different injection speeds in EAEP and EAEV under the other conditions fixing ESD at 2 cm, applied voltage at 8 kV, and SCD at 15 cm. The morphologies of nanofibers produced by both systems are round and having typical non-woven characteristics. The relationships between the fiber diameter, jet numbers, and the injection speed are shown in Figure 9. It could be found that the average diameter of nanofibers decreased with the increase of injection speed. The average diameter of nanofibers decreased from 340 nm to 250 nm in EAEV, while decreased from 290 nm to 260 nm in EAEP. Except for 2.5 mL/h, fiber diameter in EAEP was smaller than that in EAEV. One possible explanation for this might be that as injection speed increased, more liquid was being drawn out, which could be beneficial to produce multi-jets leading to finer fibers. The impact of auxiliary electrode position on the number of jets under different injection speeds is shown in Figure 9b. With the increase of the injection speed from 1 mL/h to 2.5 mL/h, the average jet number increased from 3 to 8.4 in EAEP and from 1.8 to 5.6 in EAEV, suggesting higher injection speeds gets more jets.

### 3.4. Effect of AE Position on Fiber Morphology under Different ESD

**Figure 10:**
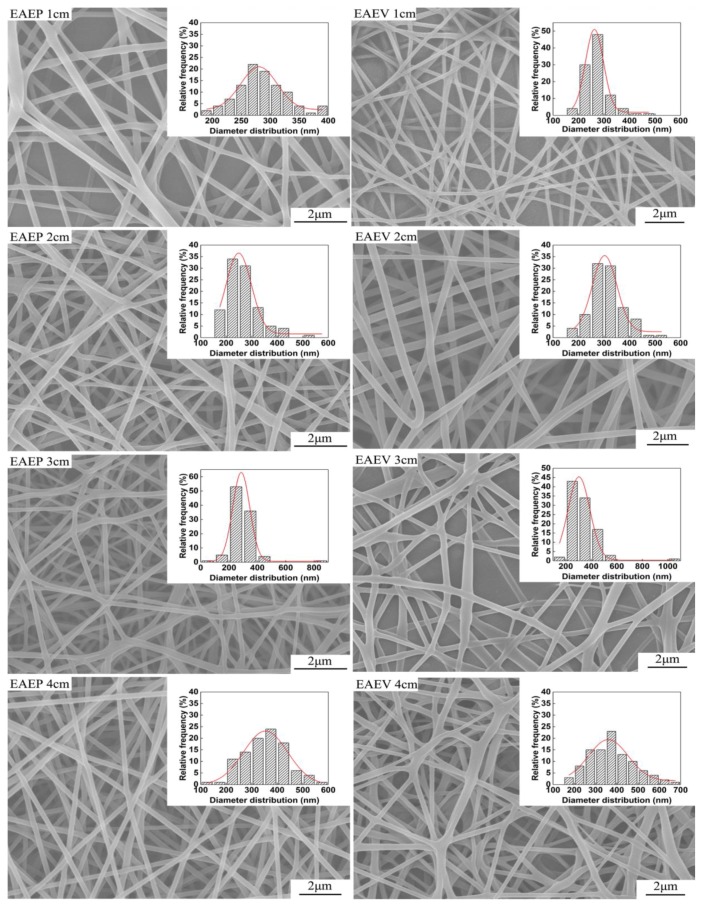
SEM images of electrospun nanofibers produced in EAEP and EAEV with different ESDs (1, 2, 3, and 4 cm).

**Figure 11:**
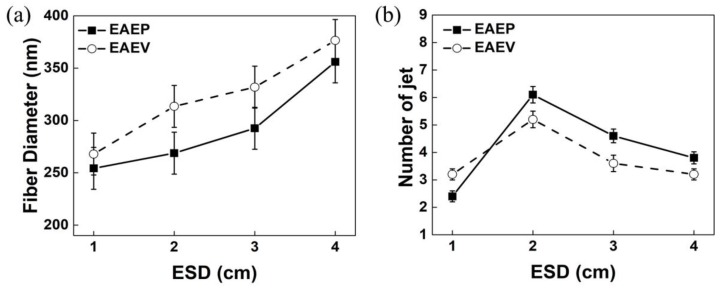
(**a**) Average fiber diameter vs. ESD and (**b**) Jet number vs. ESD in EAEP and EAEV.

In our previous work [25], it could be found that the AE position (ESD of 3–9 cm) greatly influences the fiber morphology and jet number. In order to investigate the effect of AE position on the two important factors, a closer ESD (1, 2, 3, and 4 cm) was employed under applied voltage at 8 kV, injection speed at 2 mL/h, and SCD at 15 cm. Figure 10 shows SEM images of nanofibers obtained in EAEP and EAEV. The fiber morphology is as good as shown in Figure 11a, when ESD was 2 cm, the average fiber diameter was 268 nm in EAEP, and 313 nm in EAEV showing EAEP produced thinner fibers than EAEV. One possible explanation might be that when ESD are the same, other conditions kept fixed, the electric field force produced in EAEP is stronger as shown in simulation results (Figure 5), than that in EAEV, thus leading to thinner fibers. It could conclude that the increase of ESD increased the fiber diameter. The reason might be that the increase of ESD led to the decrease of electric field intensity and hence resulting in the increase of the fiber diameter. Figure 11b shows the number of jets obtained from different ESD in EAEP and EAEV. It was found that with the decrease of ESD (4 cm **→** 1 cm), the average jet number increased, e.g., from 3.8 to 6.2 in EAEP and from 3.2 to 5.2 in EAEV. But when ESD was less than 2 cm, the jet number reduced drastically with decreasing of ESD continuously. Thoppey et al. [31] demonstrated that a stronger electric field allowed smaller spacing between jets. With the decrease of ESD, the electric field intensity became larger, and the charge density on the surface of the Taylor cone also became large, making it easy to generate multiple jets. As the ESD further decreased and the electric field intensity became larger and more concentrated, the distance of two adjacent jets gradually decreased, which suppressed the formation of jets. Thus, the number of jets increased at first and then decreased.

### 3.5. Effect of AE on the Production Rate of Nanofibers

**Figure 12:**
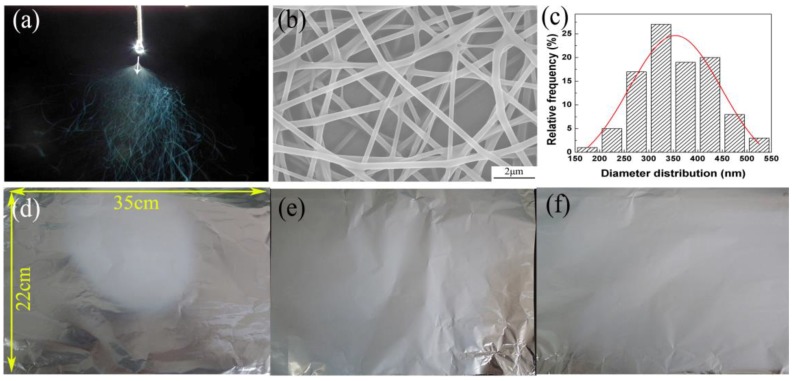
(**a**) Image of jet motion; (**b**) SEM image of nanofibers; (**c**) Diameter distribution of nanofibers produced in EWAE. The photographs of collected fiber mats (**d**) EWAE; (**e**) EAEV; (**f**) EAEP.

As shown in Figure 12, it was difficult to produce more than one jet without AE (EWAE, Figure 12a) even up to 28 kV with other conditions fixed. Figure 12b,c show SEM images of resulting PVA nanofibers and diameter distribution of the nanofibers without AE, respectively. It is worthwhile to note that PVA nanofibers without AE had the largest fiber diameter distribution of 180–510 nm, and an average diameter = 350 nm, (compared with those in EAEV, diameter distribution = 132–433 nm and average diameter = 249 nm, and EAEP, diameter distribution = 120–430 nm and average diameter = 220 nm at the same conditions). The nanofibers mat deposited on the collector within 1h was also measured to evaluate the uniformity of the deposition, as shown in Figure 12d–f. It can be found that the deposition area of mat under AE is larger than that without AE, suggesting AE can effectively improve the deposition uniformity and area.

**Figure 13:**
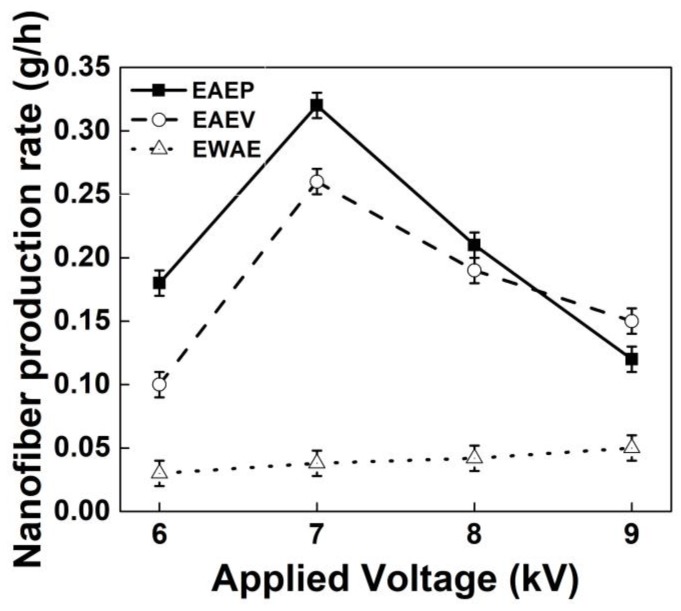
Applied voltage vs. nanofiber production rate in EAEP, EAEV and EWAE.

The production rates of nanofibers with and without AE under different applied voltages were compared and are shown in Figure 13. The results show that the nanofiber yields of EAEP can reach over 0.3 g/h, but the average yield without AE was 0.03 g/h. The nanofiber yields with AE (multiple jets in electrospinning) were 3–10 times higher than that of without AE (single jet in electrospinning) at the same condition, showing AE could enhance greatly the production rate of nanofibers.

## 4. Conclusion

In this study, electrospinning with auxiliary electrodes at parallel position (EAEP) and at vertical position (EAEV) arrangements were investigated to examine nanofiber morphology and jet number. The results showed higher electric field intensity was obtained in EAEP, which resulted in finer nanofibers (220–277 nm) with narrower fiber diameter distribution than those in EAEV. The average nanofiber diameter decreased with an increase of applied voltage and injection speed and a decrease of ESD in both EAEP and EAEV. The number of jets increased firstly and then decreased with an increase in applied voltage and ESD, respectively, while increased with an increase of injection speed. This work provides a new perspective for understanding the mechanism of electrospinning with multiple jets. It also provides an experimental basis for improving electrospinning technology and the production rate of electrospinning.
